# Intrinsic capacity as an indicator of the risk of depression and anxiety: a prospective cohort study based on the UK Biobank

**DOI:** 10.3389/fpsyg.2026.1778636

**Published:** 2026-07-27

**Authors:** Yixian Zhu, Mengtong Sun, Jianing Li, Ziqing Sun, Zexin Lou, Yujie Shi, Yichun Wu, Yuheng Yuan, Songbing Qin, Yueping Shen

**Affiliations:** 1 Department of Radiation Oncology, The First Affiliated Hospital of Soochow University, Suzhou, Jiangsu, China; 2 Department of Epidemiology and Biostatistics, School of Public Health, Medical College of Soochow University, Suzhou, Jiangsu, China; 3 Department of Infectious Diseases and Public Health, City University of Hong Kong, Hong Kong, Hong Kong SAR, China; 4 Department of Good Clinical Practice Office, The Fourth Affiliated Hospital of Soochow University, Suzhou, Jiangsu, China

**Keywords:** anxiety, depression, healthy aging, intrinsic capacity (IC), mental health

## Abstract

**Background:**

The World Health Organization has proposed the concept of intrinsic capacity (IC) as a crucial component of healthy aging. Nevertheless, prospective evidence on the association between IC and incident depression and anxiety remains limited.

**Methods:**

This cohort study involved 403,018 participants from the UK Biobank between 2006 and 2010, with 127,431 completing subsequent online follow-ups. We analyzed seven factors reflecting the functional status of four IC domains to calculate a composite IC deficit score. Depression, anxiety and depression/anxiety were assessed using hospital diagnoses and mental health questionnaires. Cox proportional hazards models and logistic regression models were employed to elucidate the relationship between IC deficit scores and the incidence of outcomes.

**Results:**

The analysis included 18,893 participants with incident depression, 22,320 participants with incident anxiety, and 76,361 participants with incident depression/anxiety, over 12.7 years of follow-up. Participants with an IC score of 4 + had a higher risk of developing depression, anxiety, and depression/anxiety than those with an IC score of 0. The hazard ratios (HRs) [95% confidence intervals (CIs)] were 3.70 (3.48–3.95), 3.01 (2.84–3.20), and 3.14 (2.98–3.32), respectively. The 7-year follow-up mental health questionnaires also confirmed this prospective relationship.

**Conclusion:**

In summary, this large prospective cohort study supports a significant association between reduced IC and a higher subsequent risk of incident depression and anxiety. Reduced IC may serve as a useful risk indicator, although further studies are needed to validate its predictive utility and to determine whether interventions targeting IC can improve mental health outcomes.

## Introduction

Depression and anxiety represent two of the most prevalent mental disorders, particularly affecting the elderly population (World Health Organization, 2017). According to the Global Burden of Disease (GBD) report in 2021, depression and anxiety ranked second (16.4%) and first (16.7%) (GBD 2021 Diseases and Injuries Collaborators, 2024), respectively, among non-communicable diseases contributing to the largest increase in age-standardized disability-adjusted life-years (DALYs) from 2010 to 2021. With the global demographic shift towards an aging population, the proportion of middle-aged and elderly individuals affected by these disorders continues to rise, underscoring the urgent need for effective risk prediction and preventive strategies (Tian et al., 2026). However, the identification of depression and anxiety remains challenging, as these conditions often remain undetectable before formal hospital diagnosis (Yang et al., 2025). This gap necessitates the development of novel risk assessment tools or health indicators to facilitate early detection and intervention.

In response to these global demographic changes and the increasing prevalence of age-related health challenges, including mental health disorders, the World Health Organization (WHO) introduced the concept of intrinsic capacity (IC) in 2015. This framework represents a shift in public health assessment, moving from a disease-oriented approach towards a focus on functional ability as a key determinant of healthy aging. IC, which encompasses physical and mental capacities, plays a crucial role in functional ability and healthy aging (Cesari et al., 2018; Beard et al., 2019). This multidimensional construct includes five domains: *locomotor*, *vitality*, *cognitive*, *psychological*, and *sensory*. This concept enables a holistic and longitudinal evaluation of health status, transcending age limitations and enabling the preemptive identification of decrements in personal capability (Stolz et al., 2022; Merchant et al., 2024; Sánchez-Sánchez et al., 2024).

Particularly concerning the geriatric population, a reduction in IC often correlates with negative health outcomes, including functional impairment, frailty, and mortality (Stolz et al., 2022; Zhou et al., 2023). Moreover, previous studies have also highlighted the associations between individual IC components and the incidence of depression and anxiety, such as low grip strength (Fukumori et al., 2015; Lee, 2024) and sleep disturbance (Baglioni et al., 2016; Fang et al., 2019). For instance, research by Gordon (Gordon et al., 2019) and Marques (Marques et al., 2020) demonstrated that low grip strength is associated with increased risk of depression and anxiety, while McCallum’s (McCallum et al., 2019) study confirmed that fatigue and sleep disturbances are also risk factors for them. Demakakos (Demakakos et al., 2013) proposed that reduced gait speed is associated with elevated depressive symptoms both concurrently and two years later. However, these studies have largely focused on the associations between individual IC components and depression or anxiety, with the relationship between IC as a composite measure and the two disorders remaining unexplored.

To address this research gap, we conducted a large-scale prospective cohort study using the UK Biobank Study to investigate longitudinal associations between IC and the risk of depression, anxiety, and depression/anxiety. The study was reported in accordance with the Strengthening the Reporting of Observational Studies in Epidemiology (STROBE) guidelines (Supplementary Table 1). The population attributable fraction (PAF) was calculated to estimate the theoretical proportion of disease risk under the assumption that all individuals had an IC score of 0. Additionally, we repeated this analysis among the subset of participants free of depression and anxiety at baseline who completed the follow-up survey to validate the prospective relationships between reduced IC and depression, anxiety, and depression/anxiety.

## Methods

### Study cohorts

The study utilized the data from the UK Biobank study, a population-based prospective study that contained 502,416 participants recruited in 2006–2010, aged 37–73 years, with large-scale follow-ups (Sudlow et al., 2015). At baseline, 22 assessment centers conducted evaluations of participants drawn from diverse settings. Data on their lifestyle and health were gathered through questionnaires, physical measures, and biological samples. Approval for UK Biobank research was granted by the North West Multicenter Research Ethics Committee. The present analyses were performed under Application Number 68136.

In this study, we sequentially excluded participants with missing components of IC (N = 51,657) and those who were lost to follow-up or with depression or anxiety at baseline (N = 47,741), resulting in a final sample of 403,018 participants for the main analysis. Of these, 127,431 participants completed an online survey, and we conducted a secondary analysis on this subgroup (Supplementary Figure 1).

### Exposures

The IC deficit score was calculated based on indicators that exemplify 4 of the 5 domains (Ramírez-Vélez et al., 2023; Sun et al., 2024). The cognitive domain was not included in the primary IC operationalization because the available baseline cognitive data had substantial missingness (>50%), and incorporating these variables into the main analysis would have markedly reduced the analytic sample and may have introduced selection bias. In the study, we utilized 7 factors to evaluate the 4 domains of IC following previous research (Cesari et al., 2018). The *psychological domain* comprised self-reported exhaustion and sleep duration. The *sensory domain* comprised vision and hearing impairment. The *vitality domain* was operationalized through grip strength and self-reported weight loss. The *locomotor domain* was measured by gait speed. The composite IC deficit score was calculated by summing the scores across the 7 factors. Details of the 7 factors and their scores are provided in Supplementary Table 2. This approach yielded a total score ranging from 0 to 7, with a score of 0 representing the optimal IC state and a score of 7 indicating the least favorable outcome. Due to the limited number of individuals with an IC deficit score of ‘6’ or ‘7’, we combined scores of 4 and above into a single category, termed ‘4 or more (4+) factors’. Subsequently, the categorical variables were transformed, with the categories assigned the values 0, 1, 2, 3, or 4 + .

### Outcomes

The diagnoses of depression and anxiety were determined according to the International Statistical Classification of Diseases and Related Health Problems, Tenth Revision (ICD-10). Specifically, individuals with documented diagnoses of depressive episode (F32) or recurrent depressive disorder (F33) were categorized as the depression group. Those meeting criteria for phobic anxiety disorders (F40) or other anxiety disorders (F41) comprised the anxiety group. Participants with any of these diagnoses were classified as the depression/anxiety group. Participants with prevalent depression or anxiety at baseline were excluded from the prospective analysis. The follow-up period began at the time of enrollment and ended with the earliest of the following events: the date of diagnosis of depression or anxiety, loss to follow-up, death, or censoring (September 16, 2022).

Furthermore, the assessments of depressive or anxious symptoms also incorporated data from the 7-year survey. Symptoms were measured by the Patient Health Questionnaire-9 (PHQ-9) (Kroenke et al., 2001) for depression and the Generalized Anxiety Disorder-7 (GAD-7) (Spitzer et al., 2006) for anxiety. Participants were required to provide a rating on a four-point Likert scale, with values ranging from 0 (none) to 3 (almost every day). In accordance with established criteria, an item was considered positive for the corresponding symptom if the item score was ≥1. A PHQ-9 score of ≥10 suggested depressive symptoms, while a GAD-7 score of ≥10 indicated anxiety symptoms (Kroenke et al., 2001; Spitzer et al., 2006). A total score of ≥10 on the PHQ-9 or GAD-7 was indicative of the presence of depressive/anxiety symptoms. The item content and scoring details for both the PHQ-9 and GAD-7 are presented in Supplementary Table 3.

### Covariates

To mitigate the potential influence of covariates that have been linked to depressive/anxiety symptoms or IC on the observed results (Fukumori et al., 2015; Gu et al., 2021), the following factors were employed as covariates in this study: demographic factors (age, sex, and ethnicity), socioeconomic status (Townsend deprivation index [TDI], education level, and annual before-tax household income), lifestyle factors (body mass index [BMI], smoking status, alcohol intake frequency, physical activity, and healthy diet score [HDS]) and chronic diseases (hypertension, diabetes, cardiovascular disease). Comprehensive descriptions for each variable are delineated in Supplementary methods.

### Statistical analysis

Descriptive statistical analyses were performed to characterize the available demographic and clinical variables for all study participants. Categorical variables were presented as the numbers and percentages, while continuous variables were expressed as means and standard deviations (SDs).

IC deficit scores were categorized into five groups: 0, 1, 2, 3, and 4+, with 0 as the reference group. Cumulative hazard curves were constructed to depict the associations between IC and the incidence of depression, anxiety, and depression/anxiety. The Cox proportional hazards model was used to investigate the associations of the IC deficit score and its components with the incidence of depression, anxiety, and depression/anxiety. The proportional hazards assumption was assessed using Schoenfeld residuals and the corresponding global test. Results are presented as hazard ratios (HRs) with 95% confidence intervals (CIs). Three models were constructed in this study. Model 1 was unadjusted. Model 2 was adjusted for age, sex, and ethnicity. Model 3 (fully adjusted model) was constructed by adding socioeconomic status, lifestyle factors, and chronic diseases to the variables in Model 2. We further performed competing-risk analyses using cause-specific Cox proportional hazards models, treating death as a competing event, to account for the possibility that death might preclude the occurrence of the outcomes of interest and to assess the robustness of the primary findings.

To evaluate the incremental predictive value of IC beyond conventional predictors, we compared a conventional model with a model additionally including IC using Harrell’s C-index, net reclassification improvement (NRI), and integrated discrimination improvement (IDI). We computed the PAF (von Cube et al., 2023) to estimate the potential reduction in the incidence risk of depression and anxiety if all individuals had an IC score of zero. To elucidate the contributions of distinct domains of IC to disease incidence, we also calculated the PAF for each domain.

We also employed logistic regression to examine the associations between IC deficit scores and depression or anxiety symptoms. Results are expressed as odds ratios (ORs) with corresponding 95% CIs. The adjustments made in this analysis were consistent with previous models.

Consequently, we conducted subgroup analyses stratified by age (<60 years; ≥60 years), sex (female; male), BMI (≤30; >30), hypertension (yes; no), diabetes (yes; no), cardiovascular disease (yes; no), and physical activity levels (low; moderate; high) to examine whether the associations varied across participant subgroups. In order to ascertain the robustness of our findings, we conducted a series of sensitivity analyses. First, to minimize the potential contribution of reverse causality to the results, we performed sensitivity analyses excluding participants who developed depression, anxiety, or depression/anxiety within the first 2 years and within the first 5 years after recruitment (Gao et al., 2023). Second, we performed a complete-case analysis by excluding participants with missing covariates. Third, to examine the potential influence of overadjustment related to BMI, we repeated the analyses after excluding BMI from the adjustment set. Fourth, to further assess potential confounding by sedentary behavior, we additionally adjusted for television viewing time and computer use time (Ramírez-Vélez et al., 2025). Fifth, to minimize potential bias arising from overlap between the *psychological* domain and early depressive manifestations, we excluded participants with baseline depressive symptoms, defined as a PHQ-2 score of 3 or higher. Sixth, to further address potential symptom overlap between the psychological IC domain and early manifestations of depression or anxiety, we recalculated the IC deficit score after removing the psychological domain, which comprised self-reported exhaustion and sleep duration, and re-estimated its associations with incident outcomes using the same fully adjusted Cox models as in the primary analysis. Finally, we performed an additional sensitivity analysis with further adjustment for baseline psychotropic medication use and chronic pain. Detailed definitions of psychotropic medication use, chronic pain, and television viewing time and computer use time are provided in the Supplementary methods.

Statistical analyses were conducted using SAS 9.4 (SAS Institute, Cary, NC, USA) and R 4.3.2. The proportion of missing data ranged from 0% for age to 22.09% for physical activity (Supplementary Table 4). Missing covariates were handled using multiple imputation with fully conditional specification implemented in SAS PROC MI. Five imputed datasets were generated, and 20 iterations were performed for each imputation. Continuous variables were imputed using linear regression, whereas categorical variables were imputed using logistic regression, as appropriate. Estimates were then combined across the imputed datasets using Rubin’s rules with PROC MIANALYZE. All *p* values were two-tailed, with statistical significance set at *p* < 0.05.

## Results

### Participant characteristics

We categorized the study population into five groups based on IC deficit scores to delineate baseline characteristics (Table 1). The mean age (SD) of the participants was 56.53 (8.10) years. Compared with individuals having an IC deficit score of 0, those with higher scores had higher mean values for age, BMI, and TDI, and increased prevalence of alcohol consumption and smoking. Conversely, annual household income, educational attainment, and HDS were lower in these groups. Baseline characteristics before imputation and in each of the five imputed datasets are presented in Supplementary Table 5. Overall, the distributions of the main variables were very similar before and after imputation.

**Table 1:** Characteristics of the study population stratified by IC deficit scores.

Characteristics	Intrinsic Capacity (IC)					
	Overall	0	1	2	3	4+
Age (y, mean ± SD)	56.53 ± 8.10	55.06 ± 8.20	56.78 ± 8.03	57.88 ± 7.83	58.62 ± 7.61	59.37 ± 7.26
Sex, *n* (%)						
Male	229,087 (45.60)	63,184 (45.75)	71,783 (48.71)	37,724 (48.29)	13,091 (45.74)	4,745 (43.88)
Female	273,329 (54.40)	74,914 (54.25)	75,586 (51.29)	40,395 (51.71)	15,528 (54.26)	6,068 (56.12)
Ethnicity, *n* (%)						
White	472,617 (94.07)	133,067 (96.60)	139,915 (95.19)	72,921 (93.67)	26,046 (91.47)	9,605 (89.33)
Others	27,921 (5.56)	4,691 (3.40)	7,064 (4.81)	4,927 (6.33)	2,434 (8.54)	1,147 (10.67)
BMI (kg/m^2^, mean ± SD)	27.43 ± 4.80	26.34 ± 4.10	27.20 ± 4.45	28.09 ± 4.90	29.22 ± 5.59	30.56 ± 6.27
Physical activity, *n* (%)						
Low	71,572 (14.25)	17,486 (15.34)	19,803 (16.70)	11,752 (19.46)	5,189 (24.85)	2,736 (37.22)
Moderate	156,518 (31.15)	47,699 (41.84)	48,271 (40.72)	24,338 (40.29)	8,203 (39.29)	2,630 (35.78)
High	157,092 (31.27)	48,806 (42.82)	50,478 (42.58)	24,308 (40.25)	7,487 (35.86)	1985 (27.00)
Drinking, *n* (%)						
Daily or almost daily	101,754 (20.25)	31,161 (22.57)	32,150 (21.83)	15,230 (19.51)	4,642 (16.24)	1,375 (12.73)
3 or 4 times a week	115,424 (22.97)	37,205 (26.95)	35,899 (24.37)	16,821 (21.54)	5,101 (17.85)	1,370 (12.69)
Once or twice a week	129,270 (25.73)	36,898 (26.73)	38,760 (26.31)	20,308 (26.01)	7,124 (24.93)	2,472 (22.89)
1–3 times a month	55,841 (11.11)	14,298 (10.35)	15,913 (10.80)	9,118 (11.68)	3,466 (12.13)	1,314 (12.17)
Special occasions only	57,997 (11.54)	11,530 (8.35)	14,856 (10.09)	9,837 (12.60)	4,623 (16.17)	2,265 (20.97)
Never	40,627 (8.09)	6,971 (5.05)	9,725 (6.60)	6,761 (8.66)	3,624 (12.68)	2004 (18.56)
Smoking, *n* (%)						
Never	273,478 (54.43)	81,391 (59.06)	81,079 (55.18)	40,960 (52.63)	14,306 (50.26)	5,054 (47.00)
Previous	173,026 (34.44)	44,282 (32.13)	51,953 (35.36)	28,673 (36.84)	10,701 (37.60)	4,070 (37.84)
Current	52,962 (10.54)	12,148 (8.81)	13,905 (9.46)	8,198 (10.53)	3,455 (12.14)	1,630 (15.16)
TDI, mean ± SD	−1.29 (3.09)	−1.78 (2.82)	−1.51 (2.96)	−1.16 (3.13)	−0.61 (3.32)	0.10 (3.51)
Education, *n* (%)						
≥College	161,130 (32.73)	53,233 (38.79)	49,910 (34.15)	22,922 (29.68)	7,008 (24.84)	1981 (18.63)
<College	331,154 (67.27)	84,015 (61.21)	96,260 (65.85)	54,311 (70.32)	21,202 (75.16)	8,652 (81.37)
Income, *n* (%)						
<£31,000	205,430 (47.62)	46,098 (37.73)	57,747 (45.00)	35,231 (52.72)	14,500 (61.00)	6,297 (72.82)
≥£31,000	225,945 (52.38)	76,092 (62.27)	70,582 (55.00)	31,595 (47.28)	9,270 (39.00)	2,350 (27.18)
Diabetes, *n* (%)						
No	475,529 (94.65)	134,705 (97.54)	141,122 (95.76)	72,675 (93.03)	25,304 (88.42)	8,748 (80.90)
Yes	26,887 (5.35)	3,393 (2.46)	6,247 (4.24)	5,444 (6.97)	3,315 (11.58)	2065 (19.10)
Hypertension, *n* (%)						
No	356,440 (70.95)	108,585 (78.63)	106,602 (72.34)	51,589 (66.04)	16,674 (58.26)	5,092 (47.09)
Yes	145,976 (29.05)	29,513 (21.37)	40,767 (27.66)	26,530 (33.96)	11,945 (41.74)	5,721 (52.91)
Cardiovascular disease, *n* (%)						
No	473,263 (94.20)	133,815 (96.90)	140,511 (95.35)	72,581 (92.91)	25,379 (88.68)	8,754 (80.96)
Yes	29,153 (5.80)	4,283 (3.10)	6,858 (4.65)	5,538 (7.09)	3,240 (11.32)	2059 (19.04)
Healthy diet score, *n* (%)						
Poor	60,995 (12.26)	15,715 (11.42)	17,675 (12.05)	9,697 (12.49)	3,772 (13.29)	1,575 (14.73)
Medium	244,460 (49.14)	67,186 (48.81)	71,003 (48.40)	38,248 (49.25)	14,200 (50.01)	5,475 (51.19)
Ideal	192,024 (38.60)	54,733 (39.77)	58,033 (39.55)	29,721 (38.27)	10,420 (36.70)	3,645 (34.08)

SD, standard deviation; IC, intrinsic capacity.

### Association between IC and depression/anxiety at the follow-up survey

The median follow-up period was 12.7 years, with an interquartile range of 10.9–14.5 years, during which 18,893 participants developed incident depression, 22,320 developed incident anxiety, and 76,361 participants developed incident depression/anxiety. Cumulative hazard curves indicate that, with a fixed IC deficit score, the risk of incident depression and anxiety escalates over time. Additionally, the cumulative incidence risk increases more rapidly over time with a higher IC deficit score (Supplementary Figure 2).

**Figure 1:**
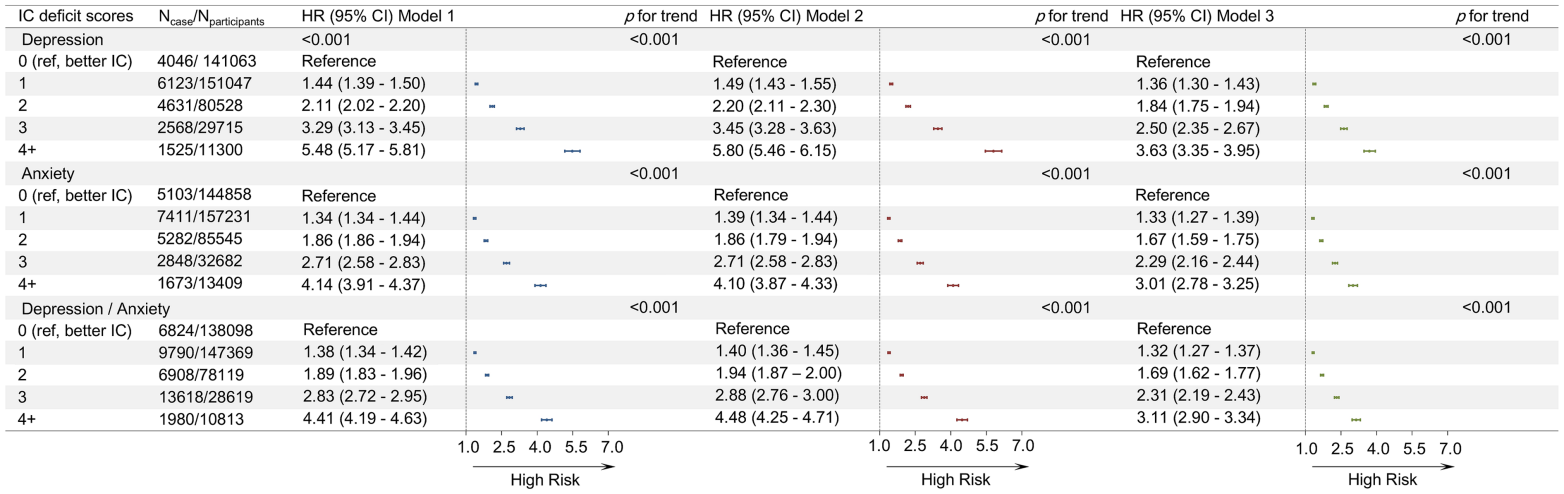
Association between IC and depression, anxiety, and depression/anxiety at follow-up. BMI, body mass index; HR, hazard ratio; IC, intrinsic capacity. Model 1 was not adjusted. Model 2 was adjusted for age, sex, and ethnicity. Model 3 was adjusted for age, sex, ethnicity, Townsend deprivation index, education level, annual before-tax household income, BMI, smoking status, alcohol intake frequency, physical activity, disease histories of hypertension status, diabetes status, and cardiovascular disease status.

Compared with participants with an IC deficit score of 0, higher IC deficit scores were associated with progressively greater risks of incident depression, anxiety, and depression/anxiety. In the fully adjusted model, the HRs (95% CIs) for depression were 1.38 (1.33–1.44), 1.88 (1.80–1.96), 2.62 (2.49–2.76), and 3.70 (3.48–3.95) for IC deficit scores of 1, 2, 3, and 4+, respectively. The corresponding HRs (95% CIs) were 1.32 (1.28–1.37), 1.68 (1.61–1.74), 2.25 (2.15–2.36), and 3.01 (2.84–3.20) for anxiety, and 1.33 (1.29–1.37), 1.71 (1.65–1.77), 2.32 (2.23–2.42), and 3.14 (2.98–3.32) for depression/anxiety, respectively (Figure 1). Moreover, we investigated the relationship between each constituent of IC and the onset of depression and anxiety, finding that all IC components were positively associated with the risk of these outcomes (Supplementary Table 6). Schoenfeld residual tests showed no evidence of violation of the proportional hazards assumption (global *p* > 0.05). In the competing-risk analysis treating death as a competing event, the associations between reduced IC and the incidence of depression, anxiety, and depression/anxiety remained materially unchanged compared with the primary analysis (Supplementary Table 7).

In a subgroup of participants who underwent a 7-year survey, we additionally validated the prospective associations of IC with the incidence of depression/anxiety, as well as with depressive/anxious symptoms. Compared with participants with an IC deficit score of 0, the ORs (95% CIs) for depression were 1.74 (1.61–1.88), 3.14 (2.90–3.41), 5.49 (4.97–6.06), and 9.12 (7.96–10.45) for IC deficit scores of 1, 2, 3, and 4+, respectively, in the fully adjusted model. The corresponding ORs (95% CIs) were 1.58 (1.46–1.70), 2.53 (2.33–2.75), 3.96 (3.58–4.39), and 6.16 (5.34–7.10) for anxiety, and 1.68 (1.59–1.79), 2.85 (2.68–3.04), 4.93 (4.56–5.33), and 8.71 (7.80–9.72) for depression/anxiety, respectively (Table 2). Additionally, analysis of data from individual questionnaire items revealed that a higher IC deficit score is associated with an increased risk of developing depressive and anxiety symptoms (Supplementary Figure 3).

**Table 2:** Associations between IC and odds of depression, anxiety and depression/anxiety at 7-year survey.

	OR (95% CI) Model 1	OR (95% CI) Model 2	OR (95% CI) Model 3
Depression			
IC deficit score			
0 (ref, better IC)	Reference	Reference	Reference
1	1.67 (1.55–1.80)	1.90 (1.77–2.05)	1.74 (1.61–1.88)
2	3.06 (2.83–3.30)	3.78 (3.49–4.09)	3.14 (2.90–3.41)
3	5.70 (5.20–6.26)	7.45 (6.77–8.19)	5.49 (4.97–6.06)
4+	11.23 (9.91–12.72)	15.37 (13.50–17.49)	9.12 (7.96–10.45)
*P* for trend	<0.001	<0.001	<0.001
Anxiety			
IC deficit score			
0 (ref, better IC)	Reference	Reference	Reference
1	1.49 (1.38–1.60)	1.65 (1.53–1.78)	1.58 (1.46–1.70)
2	2.37 (2.19–2.57)	2.79 (2.58–3.03)	2.53 (2.33–2.75)
3	3.86 (3.50–4.25)	4.65 (4.21–5.14)	3.96 (3.58–4.39)
4+	6.76 (5.92–7.72)	8.31 (7.26–9.51)	6.16 (5.34–7.10)
*P* for trend	<0.001	<0.001	<0.001
Depression/Anxiety			
IC deficit score			
0 (ref, better IC)	Reference	Reference	Reference
1	1.62 (1.53–1.71)	1.82 (1.71–1.93)	1.68 (1.59–1.79)
2	2.76 (2.60–2.93)	3.33 (3.13–3.55)	2.85 (2.68–3.04)
3	5.06 (4.69–5.44)	6.36 (5.89–6.86)	4.93 (4.56–5.33)
4+	10.29 (9.30–11.39)	13.50 (12.16–14.99)	8.71 (7.80–9.72)
*P* for trend	<0.001	<0.001	<0.001

BMI, body mass index; OR, odds ratio; IC, intrinsic capacity.

Model 1 was not adjusted. Model 2 was adjusted for age, sex, and ethnicity. Model 3 was adjusted for age, sex, ethnicity, Townsend deprivation index, education level, annual before-tax household income, BMI, smoking status, alcohol intake frequency, physical activity, disease histories of hypertension status, diabetes status and cardiovascular disease status.

### Population-attributable fractions

The result of PAF analysis (Table 3) revealed that, assuming the IC deficit score were reduced to zero among all participants, the risk of depression would decrease by 31.3% (95% CI: 29.9–33.0%). Similar reductions were observed in the anxiety and anxiety or depression groups. Specifically, 27.4% (95% CI: 25.6–29.5%) of new-onset anxiety and 27.0% (95% CI: 25.4–28.8%) of new-onset depression/anxiety could be potentially avoided if the IC score were eliminated.

**Table 3:** Population attributable fractions for depression, anxiety and depression/anxiety risk factors in the UK Biobank.

Attributable to 0 score of IC	Depression		Anxiety		Depression/anxiety	
	PAF (%)	95% CI	PAF (%)	95% CI	PAF (%)	95% CI
Model 1	38.7	(37.0–40.4)	33.4	(31.9–35.0)	33.3	(32.0–35.0)
Model 2	39.3	(37.4–41.2)	33.5	(31.8–35.4)	33.7	(32.4–35.1)
Model 3	31.3	(29.9–33.0)	27.4	(25.6–29.5)	27.0	(25.4–28.8)

BMI, body mass index; HR, hazard ratio; IC, intrinsic capacity; PAF, population-attributable fraction.

Model 1 was not adjusted. Model 2 was adjusted for age, sex, and ethnicity. Model 3 was adjusted for age, sex, ethnicity, Townsend deprivation index, education level, annual before-tax household income, BMI, smoking status, alcohol intake frequency, physical activity, disease histories of hypertension status, diabetes status and cardiovascular disease status.

Furthermore, we examined the PAF for each domain of IC. In the depression group, the PAF ranged from 7.2% (95% CI: 6.3–8.1%) in the *sensory domain* to 17.9% (95% CI: 17.1–19.1%) in the *psychological domain*. Similar results were also observed in the anxiety group and the depression/anxiety group. Comparing the PAF values across domains in different groups, the *psychological domain* (14.20, 95% CI: 13.4–15.1%) emerged as the most significant contributor to the risk of depression/anxiety (Supplementary Table 8).

### Subgroup analysis and sensitivity analysis

Subgroup analyses based on age, BMI, and hypertension status revealed significant differences in the association between reduced IC and the incidence of depression/anxiety. For instance, among individuals without hypertension, the HRs (95% CIs) of those with 4 + IC deficit scores for depression, anxiety, and depression/anxiety were 3.82 (3.49–4.19), 2.85 (2.58–3.15), and 3.21 (2.98–3.46), respectively. In contrast, among those with baseline hypertension, the HRs (95% CIs) were 3.47 (3.14–3.84), 2.70 (2.43–3.00), and 2.94 (2.72–3.19), respectively (all *P* for interaction < 0.05). The association between reduced IC and elevated incidence of depression or anxiety remained unaltered across other subgroups (*P* for interaction > 0.05) (Supplementary Tables 9–15).

The main findings were consistent across multiple sensitivity analyses. Similar associations were observed after excluding participants with baseline depressive symptoms defined by PHQ-2 ≥ 3 (Supplementary Table 16), excluding incident events occurring within the first 2 and 5 years of follow-up (Supplementary Tables 17, 18), excluding the psychological domain from the IC deficit score (Supplementary Table 19), restricting the analyses to participants with complete covariate data (Supplementary Table 20), omitting BMI from the adjustment set (Supplementary Table 21), further adjusting for television viewing time and computer use time (Supplementary Table 22), and additionally adjusting for baseline psychotropic medication use and chronic pain (Supplementary Table 23).

### Incremental predictive value of IC

We further evaluated whether IC provided incremental predictive value beyond conventional predictors. Adding IC to the conventional model yielded modest improvements in discrimination across all three outcomes. The C-index increased from 0.643 to 0.669 for depression, from 0.619 to 0.638 for anxiety, and from 0.626 to 0.648 for depression/anxiety. In addition, significant improvements were observed in risk reclassification and discrimination. The NRI (95% CI) values were 0.107 (0.080–0.139), 0.050 (0.016–0.090), and 0.076 (0.041–0.103), and the corresponding IDI (95% CI) values were 0.010 (0.006–0.014), 0.004 (0.001–0.006), and 0.008 (0.004–0.010) for depression, anxiety, and depression/anxiety, respectively (Supplementary Table 24).

## Discussion

In this large prospective study, reduced IC and its individual components were associated with higher subsequent risks of depression, anxiety, and depression/anxiety after multivariable adjustment. These associations were further supported by the 7-year follow-up analysis. They also remained materially consistent in the competing-risk analysis treating death as a competing event. The main findings remained robust across multiple sensitivity analyses. Excluding participants with baseline depressive symptoms defined by PHQ-2 ≥ 3, as well as excluding events occurring within the first 2 years and within the first 5 years of follow-up, reduced concern about potential reverse causality. When the IC deficit score was reconstructed after excluding the psychological domain, the associations with incident depression, anxiety, and depression/anxiety persisted, although the effect estimates were attenuated. In addition, the associations were materially unchanged after excluding BMI from the adjustment set and after further adjustment for television viewing time and computer use time, baseline psychotropic medication use, and chronic pain. Based on the PAF analysis, assuming that IC deficit scores were reduced to 0, the corresponding model-based estimates for depression, anxiety, and depression/anxiety were 31.3% (95% CI: 29.9–33.0%), 27.4% (95% CI: 25.6–29.5%), and 27.0% (95% CI: 25.4–28.8%), respectively. Among the IC domains, the *psychological domain* of IC was identified as the most significant contributor to the risk of new-onset depression or anxiety. This finding should be interpreted with caution because self-reported exhaustion and sleep duration may partly overlap with prodromal manifestations of these outcomes. Nevertheless, the persistence of associations in the domain-exclusion analysis suggests that the overall findings were not solely driven by the psychological domain. Overall, these findings support the potential importance of IC as a risk indicator for depression and anxiety in middle-aged and older adults.

The observed relationship between reduced IC and adverse health outcomes aligns with prior epidemiological evidence. Previous studies have tried to elucidate the underlying association linking IC components to the increased risk of depression and anxiety (Kandola et al., 2019; Cho et al., 2022; Chakrabarty et al., 2024). For instance, studies have shown that lower grip strength (Kane et al., 2016; Kandola et al., 2020; Cabanas-Sánchez et al., 2022; Chang et al., 2023) and reduced gait speed (Demakakos et al., 2013; Nazari et al., 2024) are associated with elevated risks of the two disorders. Notably, sensory impairments, particularly hearing loss (Rutherford et al., 2018; Zhang et al., 2024) and visual impairment (World Health Organization, 2019; Parravano et al., 2021), have also been shown to contribute to an elevated risk of developing depression or anxiety. However, investigations primarily focused on single factors rather than a composite assessment of IC. Our study addresses this gap by evaluating IC as a holistic measure, thereby providing a comprehensive assessment of its association with the development of depression and anxiety.

Emerging evidence suggests that the mechanisms underlying the association between IC and mental disorders may involve multiple pathways. Several studies suggest that the physical components of IC may influence mental health through pathways involving inflammatory cytokines (Liu et al., 2012; Carvalho et al., 2014; Köhler et al., 2017), chronic oxidative stress and neurocognitive factors (Firth et al., 2020; Li et al., 2023). For example, myokines (Hoffmann and Weigert, 2017), which are muscle-derived factors, have been shown to influence cognitive function and modulate mood disorders through anti-inflammatory pathways. Additionally, sleep disturbances have been shown to disrupt sleep cycles (Chellappa and Aeschbach, 2022) through neurotransmitters such as acetylcholine (Picciotto et al., 2015) and adenosine, potentially triggering or exacerbating depression and anxiety (Gujar et al., 2011; Baglioni et al., 2016; Ben Simon et al., 2020). Sensory impairment can induce pathological alterations in brain structure and function, thus diminishing cognitive reserve and impairing emotional regulation capacity (Rutherford et al., 2018; Parravano et al., 2021). These factors further compromise an individual’s social functioning (Mick et al., 2014), thereby elevating the risk of depression and anxiety.

IC captures functional status across multiple domains and may serve as an integrated marker of functional health in older adults. In this study, reduced IC was associated with higher subsequent risks of depression and anxiety. Adding IC to the conventional model yielded modest but consistent gains in discrimination and significant improvements in reclassification across all three outcomes. Nevertheless, the absolute increases in C-index were limited in magnitude, and these findings should therefore be interpreted as evidence of incremental predictive value at the population level rather than as support for immediate clinical implementation of an IC-based prediction tool. From a practical perspective, IC may help identify individuals at elevated risk of depression and anxiety, but its utility for individual-level risk prediction and clinical decision-making remains to be established. Further studies assessing model calibration, clinical utility, and external validity in diverse populations are needed to determine whether incorporating IC can meaningfully improve risk stratification in practice.

This study represents the first investigation into the association between IC and the risk of incident depression or anxiety. The large, well-characterized prospective cohort enhanced the credibility of the study, while subgroup and sensitivity analyses further supported the robustness of the findings. While this study has notable strengths, several limitations warrant careful consideration. First, depression and anxiety were identified using hospital records and self-reported questionnaires, which may have led to underascertainment of cases (Thornicroft et al., 2017). Second, we used a simple unweighted IC deficit score for ease of interpretation and consistency with previous UK Biobank-based IC studies. However, this approach may not fully reflect the relative contribution of different IC domains. In addition, we did not compare alternative weighting strategies or evaluate non-linear exposure models in the present study. These considerations may also limit direct comparisons with studies using other IC operationalizations, such as Z-score-based approaches (Sanchez-Rodriguez et al., 2024). Third, baseline cognitive data had substantial missingness, which precluded inclusion of a cognitive component in the primary IC operationalization without marked loss of sample size and possible selection bias. In a restricted sensitivity analysis including a cognitive component, the overall pattern of association remained broadly consistent with the primary findings, but this issue remains an important methodological consideration. Fourth, the UK Biobank cohort is subject to healthy volunteer bias, which may limit the generalizability of our findings. Further validation in more diverse populations is warranted. Finally, although we adjusted for a broad range of covariates and the findings remained robust in additional sensitivity analyses, residual confounding from unmeasured or imperfectly measured factors cannot be fully excluded.

## Conclusion

In conclusion, our findings support a significant prospective association between reduced IC and a higher subsequent risk of depression and anxiety. Reduced IC may serve as a useful risk indicator, although further studies are needed to validate its predictive utility and to determine whether interventions targeting IC can improve mental health outcomes.

## Data Availability

The data analyzed in this study is subject to the following licenses/restrictions: The UK Biobank dataset used in this study is not publicly available. Access is restricted to bona fide researchers who obtain permission through an approved application process. Requests to access these datasets should be directed to https://www.ukbiobank.ac.uk/.
