# Supplementary material for: Intrinsic capacity as an indicator of the risk of depression and anxiety: a prospective cohort study based on the UK Biobank
Source: Front Psychol. 2026 Jul 27;17:1778636. doi: 10.3389/fpsyg.2026.1778636 (PMC13454228; doi:10.3389/fpsyg.2026.1778636)
Supplement: Supplementary file 1 [file Data_Sheet_1.DOCX]

**Supplementary methods**

**Covariates**

In the present study, the consideration of covariates comprised four principal components: sociodemographic factors, socioeconomic status, lifestyle factors and chronic diseases. Including age, sex, and ethnicity (White, others), Townsend deprivation index [TDI], education level (none, O levels or General Certificate of Secondary Education or Certificate of Secondary Education or equivalent, A levels/AS levels or equivalent, National Vocational Qualification or Higher National Diploma or Higher National Certificate or other professional, College or University degree), annual before-tax household income (<GBP 31 000, ≥GBP 31 000), body mass index [BMI] (<18.5 kg·m^2^, 18.5–25 kg·m^2^, 25–30 kg·m^2^, ≥30 kg·m^2^), smoking status (current, former, or never), alcohol intake frequency (Never, Special occasions only, One to three times a month, Once or twice a week, Three or four times a week, Daily or almost daily), physical activity (low, moderate, and high), and healthy diet score, hypertension, diabetes status and cardiovascular disease. Townsend Deprivation Index is constructed based on four key variables in the UK Biobank: unemployment, overcrowded households, carless ownership and homeless ownership. Higher scores are indicative of elevated levels of material deprivation and under-resourcing within the respective geographical area or community. Pre-existing underlying diseases were grouped as ‘yes’ or ‘no’ for hypertension, diabetes, and cardiovascular disease based on self-reporting at baseline.

**Additional variables used in sensitivity analyses**

Television viewing time and computer use time were additionally adjusted for as indicators of sedentary behavior (1). Baseline depressive symptoms were defined as a PHQ-2 score of 3 or higher. Baseline psychotropic medication use was defined from self-reported regular medication use at baseline (UK Biobank data field 20003) (2). In UK Biobank, regular medication use was recorded at the baseline assessment based on participant self-report. Chronic pain was defined as pain in the hip, knee, back, or neck/shoulder, or pain all over the body, lasting for at least 3 months (3).

**eTable 1 STROBE checklist of cohort study**

|  | **Item No** | **Recommendation** | **Page No** |
| --- | --- | --- | --- |
| Title and abstract | 1 | (a) Indicate the study’s design with a commonly used term in the title or the abstract | 1 |
|  |  | (b) Provide in the abstract an informative and balanced summary of what was done and what was found | 2 |
| Introduction | | | |
| Background/rationale | 2 | Explain the scientific background and rationale for the investigation being reported | 5 |
| Objectives | 3 | State specific objectives, including any prespecified hypotheses | 5 |
| Methods | | | |
| Study design | 4 | Present key elements of study design early in the paper | 8-10 |
| Setting | 5 | Describe the setting, locations, and relevant dates, including periods of recruitment, exposure, follow-up, and data collection | 8 |
| Participants | 6 | (a) Give the eligibility criteria, and the sources and methods of selection of participants. Describe methods of follow-up | 8-10 |
|  |  | (b) For matched studies, give matching criteria and number of exposed and unexposed | 8-10 |
| Variables | 7 | Clearly define all outcomes, exposures, predictors, potential confounders, and effect modifiers. Give diagnostic criteria, if applicable | 8-10 |
| Data sources/ measurement | 8* | For each variable of interest, give sources of data and details of methods of assessment (measurement). Describe comparability of assessment methods if there is more than one group | 8-10 |
| Bias | 9 | Describe any efforts to address potential sources of bias | 12 |
| Study size | 10 | Explain how the study size was arrived at | 8 |
| Quantitative variables | 11 | Explain how quantitative variables were handled in the analyses. If applicable, describe which groupings were chosen and why | 8-10 |
| Statistical methods | 12 | (a) Describe all statistical methods, including those used to control for confounding | 11-12 |
|  |  | (b) Describe any methods used to examine subgroups and interactions | 12 |
|  |  | (c) Explain how missing data were addressed | 12 |
|  |  | (d) If applicable, explain how loss to follow-up was addressed | - |
|  |  | (e) Describe any sensitivity analyses | 12 |
| Results | | |  |
| Participants | 13* | (a) Report numbers of individuals at each stage of study—eg numbers potentially eligible, examined for eligibility, confirmed eligible, included in the study, completing follow-up, and analysed | 13 |
|  |  | (b) Give reasons for non-participation at each stage | 13 |
|  |  | (c) Consider use of a flow diagram | 8 |
| Descriptive data | 14* | (a) Give characteristics of study participants (eg demographic, clinical, social) and information on exposures and potential confounders | 13 |
|  |  | (b) Indicate number of participants with missing data for each variable of interest | 13 |
|  |  | (c) Summarise follow-up time (eg, average and total amount) | 13 |
| Outcome data | 15* | Report numbers of outcome events or summary measures over time | 13 |

**eTable 2 Intrinsic capacity domains and factors adapted from World Health Organization (WHO) and used in the Biobank study**

| **Domain** | **Factors** | **UK Biobank criteria** | | **UK Biobank code** |
| --- | --- | --- | --- | --- |
| Psychological | Exhaustion | Self-reported: “Over the past two weeks, how often have you felt tired or had little energy?” (Response: more than half the days, nearly every day = 1 point; not at all, several days = 0 point) | | 2080 |
|  | Sleep duration | Self-reported: “About how many hours sleep do you get in every 24 hours? (Include naps)” (Response: short 9 h/d =1; healthy 7-9 h/d = 0 points) | | 1160 |
| Sensory | Eye problems | Self-reported: non-cancer illness, self-reported considering eye/eyelid problem category (Response: yes, I am completely deaf = 1 point; no = 0 point) | | Data-Coding 6 (non-cancer Illness) Nodes: 1242, 1247, 1275, 1281, 1282, 1527, 1528, 1276, 1277, 1278, 1279, 1435, 1529, 1530, 1613 |
|  | Hearing difficulty | Self-reported: “Do you have any difficulty with your hearing?” (Response: yes, I am completely deaf = 1 point; no = 0 point) | | 2247 |
| Vitality | Weight loss | Self-reported: “Compared with one year ago, has your weight changed?” (Response: yes - lost weight = 1 point; no - weight the same, yes - gain weight = 0 point) | | 2306 |
|  | Low grip strength | Measured grip strength (sex and body-mass index adjusted cut-offs taken from Fried and colleagues) | | 31, 46, 47, 23104 |
|  |  | BMI by sex  Men  BMI ≤ 24  BMI 24.1–26  BMI 26.1–28  BMI > 28  Women  BMI ≤ 23  BMI 23.1–26  BMI 26.1–29  BMI > 29 | Cut-off (kg) criterion for frailty  ≤ 29  ≤ 30  ≤ 30  ≤ 32  ≤ 17  ≤ 17.3  ≤ 18  ≤ 21 |  |
| Locomotor | Slow walking pace | Self-reported: “How would you describe your usual walking pace?” (Response: slow pace = 1 point; steady average pace or brisk pace = 0 point) | | 924 |

**eTable 3 Items of PHQ-9 and GAD-7**

| **PHQ-9** | | | | |
| --- | --- | --- | --- | --- |
| Over the *last 2 weeks*, how often have you been bothered by any of the following problems? | Not at all | Several days | More than half the days | Nearly every day |
| 1. Little interest or pleasure in doing things | 0 | 1 | 2 | 3 |
| 2. Feeling down, depressed, or hopeless | 0 | 1 | 2 | 3 |
| 3. Trouble falling or staying asleep, or sleeping too much | 0 | 1 | 2 | 3 |
| 4. Feeling tired or having little energy | 0 | 1 | 2 | 3 |
| 5. Poor appetite or overeating | 0 | 1 | 2 | 3 |
| 6. Feeling bad about yourself—or that you are a failure or have let yourself or your family down | 0 | 1 | 2 | 3 |
| 7. Trouble concentrating on things, such as reading the newspaper or watching television | 0 | 1 | 2 | 3 |
| 8. Moving or speaking so slowly that other people could have noticed? Or the opposite—being so fidgety or restless that you have been moving around a lot more than usual | 0 | 1 | 2 | 3 |
| 9. Thoughts that you would be better off dead or of hurting yourself in some way | 0 | 1 | 2 | 3 |
| (For office coding: Total Score ____ = ____ + ____ + ____) | | | | |
| If you checked off *any* problems, how *difficult* have these problems made it for you to do your work,  take care of things at home, or get along with other people? | | | | |
|  | Not difficult at all □ | Somewhat difficult □ | Very difficult □ | Extremely difficult □ |
|  |  |  |  |  |
|  |  |  |  |  |
| **GAD-7** | | | | |
| Over the *last 2 weeks*, how often have you been bothered by any of the following problems? | Not at all | Several days | More than half the days | Nearly every day |
| 1. Feeling nervous, anxious or on edge | 0 | 1 | 2 | 3 |
| 2. Not being able to stop or control worrying | 0 | 1 | 2 | 3 |
| 3. Worrying too much about different things | 0 | 1 | 2 | 3 |
| 4. Trouble relaxing | 0 | 1 | 2 | 3 |
| 5. Being so restless that it is hard to sit still | 0 | 1 | 2 | 3 |
| 6. Becoming easily annoyed or irritable | 0 | 1 | 2 | 3 |
| 7. Feeling afraid as if something awful might happen | 0 | 1 | 2 | 3 |
| (Total Score ____ = Add Columns ____ + ____ + ____) | | | | |
| If you checked off *any* problems, how *difficult* have these problems made it for you to do your work,  take care of things at home, or get along with other people? | | | | |
|  | Not difficult at all □ | Somewhat difficult □ | Very difficult □ | Extremely difficult □ |

**eTable 4 Percentages of missing values for covariates**

| **Variable** | **The proportion of missing values** |
| --- | --- |
| Age | 0.00% |
| Sex | 0.00% |
| Ethnicity | 0.37% |
| BMI | 0.62% |
| Townsend deprivation index | 0.12% |
| Smoking status | 0.59% |
| Alcohol intake frequency | 0.30% |
| Education level | 2.02% |
| Physical activity | 23.33% |
| Income | 14.16% |
| Healthy diet score | 0.98% |
| Diabetes | 0.00% |
| Hypertension | 0.00% |
| Cardiovascular disease | 0.00% |

**eTable 5 Baseline characteristics of the study population before and after imputation**

| **Characteristic** | **Before imputation** | **After imputation** | **After imputation** | **After imputation** | **After imputation** | **After imputation** |
| --- | --- | --- | --- | --- | --- | --- |
|  |  | (the first dataset) | (the second dataset) | (the third dataset) | (the fourth dataset) | (the fifth dataset) |
| Age (y, mean±SD) | 56.02 ± 8.13 | 56.60 ± 8.10 | 56.60 ± 8.10 | 56.60 ± 8.10 | 56.60 ± 8.10 | 56.60 ± 8.10 |
| Sex(n, %) |  |  |  |  |  |  |
| Male | 140,156 (49.24) | 212,491 (52.72) | 212,491 (52.72) | 212,491 (52.72) | 212,491 (52.72) | 212,491 (52.72) |
| Female | 144,492 (50.76) | 190,527 (47.28) | 190,527 (47.28) | 190,527 (47.28) | 190,527 (47.28) | 190,527 (47.28) |
| Ethnicity, n (%) |  |  |  |  |  |  |
| White | 272,234 (95.64) | 382,643 (94.94) | 382,641 (94.94) | 382,643 (94.94) | 382,634 (94.94) | 382,636 (94.94) |
| Others | 12,414 (4.36) | 20,375 (5.06) | 20,377 (5.06) | 20,375 (5.06) | 20,384 (5.06) | 20,382 (5.06) |
| BMI (kg/m2, mean ± SD) | 27.18 ± 4.58 | 27.31 ± 4.68 | 27.31 ± 4.68 | 27.31 ± 4.68 | 27.31 ± 4.68 | 27.31 ± 4.68 |
| Physical activity, n (%) |  |  |  |  |  |  |
| Low | 50,854 (17.87) | 72,782 (18.06) | 72,856 (18.08) | 72,644 (18.03) | 72,799 (18.06) | 72,915 (18.09) |
| Moderate | 117,082 (41.13) | 164,098 (40.72) | 164,021 (40.70) | 164,102 (40.72) | 163,930 (40.68) | 164,104 (40.72) |
| High | 116,712 (41.00) | 166,138 (41.22) | 166,141 (41.22) | 166,272 (41.26) | 166,289 (41.26) | 165,999 (41.19) |
| Smoking, n (%) |  |  |  |  |  |  |
| Never | 157,702 (55.40) | 223,430 (55.44) | 223,393 (55.43) | 223,401 (55.43) | 223,375 (55.43) | 223,387 (55.43) |
| Previous | 99,920 (35.10) | 140,107 (34.76) | 140,153 (34.78) | 140,146 (34.77) | 140,168 (34.78) | 140,145 (34.77) |
| Current | 27,026 (9.49) | 39,481 (9.80) | 39,472 (9.79) | 39,471 (9.79) | 39,475 (9.79) | 39,486 (9.80) |
| TDI, mean ± SD | -1.51 ± 2.97 | -1.43 ± 3.02 | -1.43 ± 3.02 | -1.43 ± 3.02 | -1.43 ± 3.02 | -1.43 ± 3.02 |
| Education (%) |  |  |  |  |  |  |
| ≥College | 109,116 (38.33) | 136,006 (33.75) | 136,003 (33.75) | 135,999 (33.75) | 135,995 (33.74) | 135,992 (33.74) |
| <College | 175,532 (61.67) | 267,012 (66.25) | 267,015 (66.25) | 267,019 (66.25) | 267,023 (66.26) | 267,026 (66.26) |
| Income, n (%) |  |  |  |  |  |  |
| <£31,000 | 122,443 (43.02) | 191,404 (47.49) | 191,625 (47.55) | 191,424 (47.50) | 191,399 (47.49) | 191,325 (47.47) |
| ≥£31,000 | 162,205 (56.98) | 211,614 (52.51) | 211,393 (52.45) | 211,594 (52.50) | 211,619 (52.51) | 211,693 (52.53) |
| Drinking, n (%) |  |  |  |  |  |  |
| Daily or almost daily | 63,986 (22.48) | 84,591 (20.99) | 84,588 (20.99) | 84,591 (20.99) | 84,596 (20.99) | 84,593 (20.99) |
| 3 or 4 times a week | 71,821 (25.23) | 96,430 (23.93) | 96,434 (23.93) | 96,428 (23.93) | 96,425 (23.93) | 96,435 (23.93) |
| Once or twice a week | 73,825 (25.94) | 105,605 (26.20) | 105,610 (26.20) | 105,603 (26.20) | 105,598 (26.20) | 105,603 (26.20) |
| 1–3 times a month | 30,395 (10.68) | 44,130 (10.95) | 44,132 (10.95) | 44,133 (10.95) | 44,134 (10.95) | 44,133 (10.95) |
| Special occasions only | 27,005 (9.49) | 43,136 (10.70) | 43,137 (10.70) | 43,139 (10.70) | 43,138 (10.70) | 43,133 (10.70) |
| Never | 17,616 (6.19) | 29,126 (7.23) | 29,117 (7.22) | 29,124 (7.23) | 29,127 (7.23) | 29,121 (7.23) |
| Healthy diet score, n (%) |  |  |  |  |  |  |
| Poor | 34,165 (12.00) | 48,769 (12.10) | 48,757 (12.10) | 48,786 (12.11) | 48,781 (12.10) | 48,774 (12.10) |
| Medium | 138,346 (48.60) | 197,103 (48.91) | 197,093 (48.90) | 197,073 (48.90) | 197,105 (48.91) | 197,072 (48.90) |
| Ideal | 112,137 (39.39) | 157,146 (38.99) | 157,168 (39.00) | 157,159 (39.00) | 157,132 (38.99) | 157,172 (39.00) |
| Diabetes, n (%) |  |  |  |  |  |  |
| No | 271,326 (95.32) | 382,554 (94.92) | 382,554 (94.92) | 382,554 (94.92) | 382,554 (94.92) | 382,554 (94.92) |
| Yes | 13,322 (4.68) | 20,464 (5.08) | 20,464 (5.08) | 20,464 (5.08) | 20,464 (5.08) | 20,464 (5.08) |
| Hypertension, n (%) |  |  |  |  |  |  |
| No | 207,567 (72.92) | 288,542 (71.60) | 288,542 (71.60) | 288,542 (71.60) | 288,542 (71.60) | 288,542 (71.60) |
| Yes | 77,081 (27.08) | 114,476 (28.40) | 114,476 (28.40) | 114,476 (28.40) | 114,476 (28.40) | 114,476 (28.40) |
| Cardiovascular disease, n (%) |  |  |  |  |  |  |
| No | 270,271 (94.95) | 381,040 (94.55) | 381,040 (94.55) | 381,040 (94.55) | 381,040 (94.55) | 381,040 (94.55) |
| Yes | 14,377 (5.05) | 21,978 (5.45) | 21,978 (5.45) | 21,978 (5.45) | 21,978 (5.45) | 21,978 (5.45) |

SD, standard deviation; IC, intrinsic capacity.

**eTable 6 Association between each factor of IC and depression, anxiety and depression/anxiety**

| **Components of IC factors** | **Depression** | | | **Anxiety** | | | **Depression/Anxiety** | | |
| --- | --- | --- | --- | --- | --- | --- | --- | --- | --- |
|  | **N_case_/N_participants_** | **HR (95% CI)** | ***P* for trend** | **N_case_/N_participants_** | **HR (95% CI)** | ***P* for trend** | **N_case_/N_participants_** | **HR (95% CI)** | ***P* for trend** |
| **Exhaustion** |  |  | <0.001 |  |  | <0.001 |  |  | <0.001 |
| 0 | 14443/369155 | Reference |  | 17249/382580 | Reference |  | 23122/360454 | Reference |  |
| 1 | 4450/44498 | 2.14 (2.06-2.21) |  | 5071/51145 | 1.93 (1.87-2.00) |  | 5998/42564 | 1.93 (1.88-1.99) |  |
|  |  |  |  |  |  |  |  |  |  |
| **Sleep duration** |  |  | <0.001 |  |  | <0.001 |  |  | <0.001 |
| 0 | 12431/307855 | Reference |  | 15006/320911 | Reference |  | 19600/300290 | Reference |  |
| 1 | 6462/105798 | 1.37 (1.32-1.41) |  | 7314/112814 | 1.29 (1.25-1.33) |  | 9520/102728 | 1.32 (1.29-1.35) |  |
|  |  |  |  |  |  |  |  |  |  |
| **Eye/eyelid problems** |  |  | <0.001 |  |  | <0.001 |  |  | <0.001 |
| 0 | 17684/391232 | Reference |  | 20742/409878 | Reference |  | 27176/381291 | Reference |  |
| 1 | 1209/22421 | 1.24 (1.17-1.31) |  | 1578/23847 | 1.34 (1.28-1.41) |  | 1944/21727 | 1.30 (1.24-1.36) |  |
|  |  |  |  |  |  |  |  |  |  |
| **Hearing impairment** |  |  | <0.001 |  |  | <0.001 |  |  | <0.001 |
| 0 | 13283/309767 | Reference |  | 15841/323632 | Reference |  | 20738/302102 | Reference |  |
| 1 | 5610/103886 | 1.28 (1.24-1.32) |  | 6479/110093 | 1.24 (1.21-1.28) |  | 8382/100916 | 1.24 (1.21-1.28) |  |
|  |  |  |  |  |  |  |  |  |  |
| **Weight loss** |  |  | <0.001 |  |  | <0.001 |  |  | <0.001 |
| 0 | 15219/351400 | Reference |  | 18233/367552 | Reference |  | 23766/342545 | Reference |  |
| 1 | 3674/62253 | 1.27 (1.22-1.31) |  | 4087/66173 | 1.19 (1.15-1.23) |  | 5354/60473 | 1.21 (1.17-1.25) |  |
|  |  |  |  |  |  |  |  |  |  |
| **Low grip strength** |  |  | <0.001 |  |  | <0.001 |  |  | <0.001 |
| 0 | 13526/332878 | Reference |  | 16173/347376 | Reference |  | 21284/324677 | Reference |  |
| 1 | 5367/80775 | 1.42 (1.37-1.47) |  | 6147/86349 | 1.33 (1.29-1.37) |  | 7836/78341 | 1.34 (1.30-1.38) |  |
|  |  |  |  |  |  |  |  |  |  |
| **Slow walking pace** |  |  | <0.001 |  |  | <0.001 |  |  | <0.001 |
| 0 | 15969/383875 | Reference |  | 19207/400350 | Reference |  | 25157/374257 | Reference |  |
| 1 | 2924/29778 | 1.73 (1.66-1.81) |  | 3113/33375 | 1.57 (1.50-1.64) |  | 3963/28761 | 1.62 (1.56-1.68) |  |

HR, hazard ratio; CI, confidence interval; IC, intrinsic capacity.

The model was adjusted for age, sex, ethnicity, Townsend deprivation index, education level, annual before-tax household income, BMI, smoking status, alcohol intake frequency, physical activity, disease histories of hypertension status, diabetes status and cardiovascular disease status.

**eTable 7** **Competing-risk analysis of the associations between IC and risk of depression, anxiety, and depression/anxiety with death treated as a competing event**

| **IC deficit scores** | **N_case_/N_participants_** | **HR (95% CI)** | ***P* for trend** |
| --- | --- | --- | --- |
| **Depression** | |  | <0.001 |
| 0 (ref, better IC) | 3715/138098 | Reference |  |
| 1 | 5606/147369 | 1.38 (1.33-1.44) | |
| 2 | 4189/78119 | 1.87 (1.79-1.96) | |
| 3 | 2326/28619 | 2.66 (2.52-2.80) | |
| 4+ | 1389/10813 | 3.87 (3.63-4.14) | |
| **Anxiety** |  |  | <0.001 |
| 0 (ref, better IC) | 4216/138098 | Reference |  |
| 1 | 5933/147369 | 1.31 (1.26-1.36) | |
| 2 | 4026/78119 | 1.63 (1.56-1.71) | |
| 3 | 2049/28619 | 2.18 (2.07-2.31) | |
| 4+ | 1078/10813 | 2.93 (2.73-3.14) | |
| **Depression/Anxiety** | |  | <0.001 |
| 0 (ref, better IC) | 6824/138098 | Reference |  |
| 1 | 9790/147369 | 1.33 (1.29-1.37) | |
| 2 | 6908/78119 | 1.72 (1.66-1.78) | |
| 3 | 3618/28619 | 2.35 (2.26-2.46) | |
| 4+ | 1980/10813 | 3.25 (3.08-3.43) | |

HR, hazard ratio; CI, confidence interval; IC, intrinsic capacity.

The model was adjusted for age, sex, ethnicity, Townsend deprivation index, education level, annual before-tax household income, BMI, smoking status, alcohol intake frequency, physical activity, disease histories of hypertension status, diabetes status and cardiovascular disease status.

**eTable 8 Population attributable fractions for depression, anxiety and depression/anxiety risk factors in the UK Biobank**

|  | **Depression** | **Anxiety** | **Depression/Anxiety** |
| --- | --- | --- | --- |
|  | **PAF (95% CI)** | **PAF (95% CI)** | **PAF (95% CI)** |
| Attributable to 0 score of the *Psychological* domain | 17.9 (17.1-19.1) | 14.2 (13.1-15.2) | 14.2 (13.4-15.1) |
| Attributable to 0 score of the *Sensory* domain | 7.2 (6.3-8.1) | 7.5 (6.5-8.6) | 6.6 (5.7-7.4) |
| Attributable to 0 score of the *Vitality* domain | 11.6 (10.4-12.6) | 8.2 (7.5-9.2) | 7.8 (6.9-8.7) |
| Attributable to 0 score of the *Locomotor* domain | 10.3 (9.2-11.2) | 7.1 (5.8-8.4) | 7.0 (6.0-7.7) |

PAF, population attributable fraction; CI, confidence interval; IC, intrinsic capacity.

The model was adjusted for age, sex, ethnicity, Townsend deprivation index, education level, annual before-tax household income, BMI, smoking status, alcohol intake frequency, physical activity, disease histories of hypertension status, diabetes status, cardiovascular disease status.

**eTable 9 Subgroup analyses for the association between IC and risk of depression, anxiety and depression/anxiety stratified by age**

|  | **Depression** | | | **Anxiety** | | | **Depression/Anxiety** | | |
| --- | --- | --- | --- | --- | --- | --- | --- | --- | --- |
|  | **Age<60** | **Age≥60** | ***P* for interaction** | **Age<60** | **Age≥60** | ***P* for interaction** | **Age<60** | **Age≥60** | ***P* for interaction** |
|  | **HR (95% CI)** | **HR (95% CI)** |  | **HR (95% CI)** | **HR (95% CI)** |  | **HR (95% CI)** | **HR (95% CI)** |  |
| IC deficit score |  |  | <0.001 |  |  | 0.108 |  |  | <0.001 |
| 0 score | Reference | Reference |  | Reference | Reference |  | Reference | Reference |  |
| 1 score | 1.38 (1.31-1.46) | 1.32 (1.24-1.42) |  | 1.31 (1.24-1.38) | 1.28 (1.20-1.36) |  | 1.33 (1.28-1.38) | 1.29 (1.23-1.35) |  |
| 2 scores | 1.91 (1.80-2.02) | 1.72 (1.60-1.84) |  | 1.64 (1.55-1.75) | 1.56 (1.46-1.67) |  | 1.75 (1.67-1.83) | 1.61 (1.53-1.70) |  |
| 3 scores | 2.77 (2.58-2.97) | 2.34 (2.16-2.55) |  | 2.16 (2-2.34) | 2.10 (1.94-2.27) |  | 2.39 (2.26-2.54) | 2.18 (2.05-2.32) |  |
| 4+ scores | 3.73 (3.41-4.09) | 3.61 (3.27-3.97) |  | 2.84 (2.56-3.14) | 2.79 (2.53-3.08) |  | 3.18 (2.95-3.43) | 3.05 (2.83-3.29) |  |

HR, hazard ratio; CI, confidence interval; IC, intrinsic capacity.

The model was adjusted for sex, ethnicity, Townsend deprivation index, education level, annual before-tax household income, BMI, smoking status, alcohol intake frequency, physical activity, disease histories of hypertension status, diabetes status and cardiovascular disease status.

IC scores were assessed by seven factors that reflected functional status to compute a comprehensive IC deficit score, yielding a potential maximum of 7, indicative of the poorest IC, and a minimum of 0, denoting optimal IC. The categorical variable was transformed to create discrete score, categorized as 0, 1, 2, 3, or 4+ score.

**eTable 10 Subgroup analyses for the association between IC and risk of depression, anxiety and depression/anxiety stratified by sex**

|  | **Depression** | | | **Anxiety** | | | **Depression/Anxiety** | | |
| --- | --- | --- | --- | --- | --- | --- | --- | --- | --- |
|  | **Female** | **Male** | ***P* for interaction** | **Female** | **Male** | ***P* for interaction** | **Female** | **Male** | ***P* for interaction** |
|  | **HR (95% CI)** | **HR (95% CI)** |  | **HR (95% CI)** | **HR (95% CI)** |  | **HR (95% CI)** | **HR (95% CI)** |  |
| IC deficit score |  |  | 0.057 |  |  | 0.163 |  |  | 0.069 |
| 0 score | Reference | Reference |  | Reference | Reference |  | Reference | Reference |  |
| 1 score | 1.40 (1.33-1.48) | 1.32 (1.24-1.42) |  | 1.30 (1.24-1.36) | 1.30 (1.22-1.40) |  | 1.32 (1.27-1.38) | 1.31 (1.25-1.38) |  |
| 2 scores | 1.82 (1.72-1.93) | 1.87 (1.74-2.00) |  | 1.65 (1.56-1.74) | 1.55 (1.44-1.68) |  | 1.70 (1.63-1.77) | 1.69 (1.60-1.79) |  |
| 3 scores | 2.60 (2.43-2.78) | 2.56 (2.35-2.79) |  | 2.10 (1.96-2.25) | 2.22 (2.02-2.44) |  | 2.27 (2.15-2.39) | 2.36 (2.21-2.53) |  |
| 4+ scores | 3.65 (3.35-3.97) | 3.81 (3.42-4.23) |  | 2.79 (2.55-3.04) | 2.91 (2.58-3.29) |  | 3.05 (2.86-3.26) | 3.28 (3.00-3.58) |  |

HR, hazard ratio; CI, confidence interval; IC, intrinsic capacity.

The model was adjusted for age, ethnicity, Townsend deprivation index, education level, annual before-tax household income, BMI, smoking status, alcohol intake frequency, physical activity, disease histories of hypertension status, diabetes status and cardiovascular disease status.

IC scores were assessed by seven factors that reflected functional status to compute a comprehensive IC deficit score, yielding a potential maximum of 7, indicative of the poorest IC, and a minimum of 0, denoting optimal IC. The categorical variable was transformed to create discrete score, categorized as 0, 1, 2, 3, or 4+ score.

**eTable 11 Subgroup analyses for the association between IC and risk of depression, anxiety and depression/anxiety stratified by BMI**

|  | **Depression** | | | **Anxiety** | | | **Depression/Anxiety** | | |
| --- | --- | --- | --- | --- | --- | --- | --- | --- | --- |
|  | **BMI≤30** | **BMI>30** | ***P* for interaction** | **BMI≤30** | **BMI>30** | ***P* for interaction** | **BMI≤30** | **BMI>30** | ***P* for interaction** |
|  | **HR (95% CI)** | **HR (95% CI)** |  | **HR (95% CI)** | **HR (95% CI)** |  | **HR (95% CI)** | **HR (95% CI)** |  |
| IC deficit score |  |  | 0.106 |  |  | 0.026 |  |  | 0.104 |
| 0 score | Reference | Reference |  | Reference | Reference |  | Reference | Reference |  |
| 1 score | 1.36 (1.30-1.43) | 1.37 (1.25-1.49) |  | 1.28 (1.23-1.34) | 1.35 (1.23-1.47) |  | 1.30 (1.26-1.35) | 1.36 (1.27-1.46) |  |
| 2 scores | 1.81 (1.72-1.91) | 1.85 (1.69-2.02) |  | 1.60 (1.52-1.68) | 1.63 (1.49-1.79) |  | 1.68 (1.61-1.74) | 1.73 (1.61-1.86) |  |
| 3 scores | 2.57 (2.41-2.75) | 2.57 (2.33-2.84) |  | 2.21 (2.07-2.36) | 2.03 (1.83-2.26) |  | 2.31 (2.19-2.43) | 2.31 (2.13-2.49) |  |
| 4+ scores | 3.83 (3.51-4.19) | 3.60 (3.23-4.01) |  | 2.89 (2.64-3.18) | 2.79 (2.47-3.14) |  | 3.20 (2.98-3.43) | 3.13 (2.86-3.41) |  |

HR, hazard ratio; CI, confidence interval; IC, intrinsic capacity; BMI, body mass index.

The model was adjusted for age, sex, ethnicity, Townsend deprivation index, education level, annual before-tax household income, smoking status, alcohol intake frequency, physical activity, disease histories of hypertension status, diabetes status and cardiovascular disease status.

IC scores were assessed by seven factors that reflected functional status to compute a comprehensive IC deficit score, yielding a potential maximum of 7, indicative of the poorest IC, and a minimum of 0, denoting optimal IC. The categorical variable was transformed to create discrete score, categorized as 0, 1, 2, 3, or 4+ score.

**eTable 12 Subgroup analyses for the association between IC and risk of depression, anxiety and depression/anxiety stratified by hypertension**

|  | **Depression** | | | **Anxiety** | | | **Depression/Anxiety** | | |
| --- | --- | --- | --- | --- | --- | --- | --- | --- | --- |
|  | **Yes** | **No** | ***P* for interaction** | **Yes** | **No** | ***P* for interaction** | **Yes** | **No** | ***P* for interaction** |
|  | **HR (95% CI)** | **HR (95% CI)** |  | **HR (95% CI)** | **HR (95% CI)** |  | **HR (95% CI)** | **HR (95% CI)** |  |
| IC deficit score |  |  | 0.020 |  |  | <0.001 |  |  | <0.001 |
| 0 score | Reference | Reference |  | Reference | Reference |  | Reference | Reference |  |
| 1 score | 1.29 (1.19-1.39) | 1.39 (1.33-1.46) |  | 1.24 (1.15-1.33) | 1.32 (1.26-1.38) |  | 1.25 (1.18-1.32) | 1.34 (1.29-1.39) |  |
| 2 scores | 1.70 (1.57-1.85) | 1.89 (1.79-1.99) |  | 1.45 (1.34-1.57) | 1.68 (1.60-1.78) |  | 1.55 (1.46-1.65) | 1.75 (1.68-1.82) |  |
| 3 scores | 2.40 (2.20-2.63) | 2.65 (2.48-2.84) |  | 1.92 (1.75-2.10) | 2.26 (2.11-2.42) |  | 2.10 (1.96-2.25) | 2.40 (2.28-2.53) |  |
| 4+ scores | 3.47 (3.14-3.84) | 3.82 (3.49-4.19) |  | 2.70 (2.43-3.00) | 2.85 (2.58-3.15) |  | 2.94 (2.72-3.19) | 3.21 (2.98-3.46) |  |

HR, hazard ratio; CI, confidence interval; IC, intrinsic capacity.

The model was adjusted for age, sex, ethnicity, Townsend deprivation index, education level, annual before-tax household income, BMI, smoking status, alcohol intake frequency, physical activity, disease histories of diabetes status and cardiovascular disease status.

IC scores were assessed by seven factors that reflected functional status to compute a comprehensive IC deficit score, yielding a potential maximum of 7, indicative of the poorest IC, and a minimum of 0, denoting optimal IC. The categorical variable was transformed to create discrete score, categorized as 0, 1, 2, 3, or 4+ score.

**eTable 13 Subgroup analyses for the association between IC and risk of depression, anxiety and depression/anxiety stratified by diabetes**

|  | **Depression** | | | **Anxiety** | | | **Depression/Anxiety** | | |
| --- | --- | --- | --- | --- | --- | --- | --- | --- | --- |
|  | **Yes** | **No** | ***P* for interaction** | **Yes** | **No** | ***P* for interaction** | **Yes** | **No** | ***P* for interaction** |
|  | **HR (95% CI)** | **HR (95% CI)** |  | **HR (95% CI)** | **HR (95% CI)** |  | **HR (95% CI)** | **HR (95% CI)** |  |
| IC deficit score |  |  | 0.052 |  |  | 0.055 |  |  | 0.124 |
| 0 score | Reference | Reference |  | Reference | Reference |  | Reference | Reference |  |
| 1 score | 1.22 (0.98-1.50) | 1.38 (1.32-1.44) |  | 1.40 (1.12-1.74) | 1.29 (1.24-1.35) |  | 1.25 (1.06-1.47) | 1.32 (1.28-1.36) |  |
| 2 scores | 1.85 (1.51-2.28) | 1.83 (1.75-1.92) |  | 1.51 (1.20-1.88) | 1.62 (1.55-1.69) |  | 1.61 (1.37-1.90) | 1.70 (1.64-1.76) |  |
| 3 scores | 2.37 (1.91-2.93) | 2.60 (2.45-2.75) |  | 1.79 (1.42-2.27) | 2.18 (2.06-2.30) |  | 2.08 (1.75-2.46) | 2.32 (2.22-2.42) |  |
| 4+ scores | 3.09 (2.47-3.86) | 3.82 (3.56-4.10) |  | 2.53 (1.98-3.24) | 2.86 (2.66-3.09) |  | 2.63 (2.20-3.14) | 3.21 (3.04-3.40) |  |

HR, hazard ratio; CI, confidence interval; IC, intrinsic capacity.

The model was adjusted for age, sex, ethnicity, Townsend deprivation index, education level, annual before-tax household income, BMI, smoking status, alcohol intake frequency, physical activity, disease histories of hypertension status and cardiovascular disease status.

IC scores were assessed by seven factors that reflected functional status to compute a comprehensive IC deficit score, yielding a potential maximum of 7, indicative of the poorest IC, and a minimum of 0, denoting optimal IC. The categorical variable was transformed to create discrete score, categorized as 0, 1, 2, 3, or 4+ score.

**eTable 14 Subgroup analyses for the association between IC and risk of depression, anxiety and depression/anxiety stratified by cardiovascular disease**

|  | **Depression** | | | **Anxiety** | | | **Depression/Anxiety** | | |
| --- | --- | --- | --- | --- | --- | --- | --- | --- | --- |
|  | **Yes** | **No** | ***P* for interaction** | **Yes** | **No** | ***P* for interaction** | **Yes** | **No** | ***P* for interaction** |
|  | **HR (95% CI)** | **HR (95% CI)** |  | **HR (95% CI)** | **HR (95% CI)** |  | **HR (95% CI)** | **HR (95% CI)** |  |
| IC deficit score |  |  | 0.155 |  |  | 0.756 |  |  | 0.231 |
| 0 score | Reference | Reference |  | Reference | Reference |  | Reference | Reference |  |
| 1 score | 1.27 (1.06-1.53) | 1.37 (1.32-1.43) |  | 1.37 (1.13-1.67) | 1.30 (1.24-1.35) |  | 1.33 (1.15-1.53) | 1.32 (1.28-1.36) |  |
| 2 scores | 1.73 (1.44-2.08) | 1.84 (1.75-1.93) |  | 1.66 (1.37-2.02) | 1.61 (1.54-1.68) |  | 1.64 (1.42-1.90) | 1.70 (1.64-1.76) |  |
| 3 scores | 2.33 (1.92-2.83) | 2.60 (2.46-2.76) |  | 2.18 (1.77-2.68) | 2.14 (2.02-2.27) |  | 2.24 (1.92-2.61) | 2.31 (2.21-2.41) |  |
| 4+ scores | 3.75 (3.49-4.03) | 3.40 (2.78-4.16) |  | 3.19 (2.56-3.97) | 2.79 (2.58-3.01) |  | 3.04 (2.59-3.58) | 3.16 (2.98-3.34) |  |

HR, hazard ratio; CI, confidence interval; IC, intrinsic capacity.

The model was adjusted for age, sex, ethnicity, Townsend deprivation index, education level, annual before-tax household income, smoking status, alcohol intake frequency, physical activity, disease histories of hypertension status and diabetes status.

IC scores were assessed by seven factors that reflected functional status to compute a comprehensive IC deficit score, yielding a potential maximum of 7, indicative of the poorest IC, and a minimum of 0, denoting optimal IC. The categorical variable was transformed to create discrete score, categorized as 0, 1, 2, 3, or 4+ score.

**eTable 15 Subgroup analyses for the association between IC and risk of depression, anxiety and depression/anxiety stratified by physical activity**

|  | **Depression** | | |  | **Anxiety** | | |  | **Depression/Anxiety** | | |  |
| --- | --- | --- | --- | --- | --- | --- | --- | --- | --- | --- | --- | --- |
|  | **Low** | **Moderate** | **High** | ***P* for interaction** | **Low** | **Moderate** | **High** | ***P* for interaction** | **Low** | **Moderate** | **High** | ***P* for interaction** |
|  | **HR (95% CI)** | **HR (95% CI)** | **HR (95% CI)** |  | **HR (95% CI)** | **HR (95% CI)** | **HR (95% CI)** |  | **HR (95% CI)** | **HR (95% CI)** | **HR (95% CI)** |  |
| IC deficit score |  |  |  |  |  |  |  |  |  |  |  |  |
| 0 score | Reference | Reference | Reference | 0.408 | Reference | Reference | Reference | 0.309 | Reference | Reference | Reference | 0.561 |
| 1 score | 1.37 (1.24-1.51) | 1.37 (1.28-1.47) | 1.37 (1.29-1.46) |  | 1.30 (1.18-1.43) | 1.32 (1.24-1.40) | 1.29 (1.21-1.37) |  | 1.32 (1.23-1.42) | 1.32 (1.26-1.39) | 1.32 (1.26-1.38) |  |
| 2 scores | 1.96 (1.77-2.18) | 1.82 (1.69-1.95) | 1.80 (1.68-1.94) |  | 1.55 (1.40-1.72) | 1.66 (1.55-1.78) | 1.60 (1.50-1.72) |  | 1.72 (1.59-1.86) | 1.71 (1.62-1.81) | 1.67 (1.59-1.76) |  |
| 3 scores | 2.84 (2.53-3.18) | 2.61 (2.39-2.84) | 2.42 (2.22-2.65) |  | 2.24 (1.99-2.51) | 2.18 (2.00-2.38) | 2.04 (1.86-2.23) |  | 2.44 (2.23-2.67) | 2.34 (2.19-2.50) | 2.19 (2.04-2.34) |  |
| 4+ scores | 3.78 (3.32-4.29) | 3.94 (3.54-4.39) | 3.51 (3.13-3.94) |  | 2.93 (2.56-3.35) | 3.01 (2.68-3.37) | 2.55 (2.25-2.90) |  | 3.23 (2.91-3.57) | 3.26 (2.99-3.56) | 2.95 (2.69-3.25) |  |

HR, hazard ratio; CI, confidence interval; IC, intrinsic capacity.

The model was adjusted for age, sex, ethnicity, Townsend deprivation index, education level, annual before-tax household income, smoking status, alcohol intake frequency, disease histories of hypertension status and cardiovascular disease status.

IC scores were assessed by seven factors that reflected functional status to compute a comprehensive IC deficit score, yielding a potential maximum of 7, indicative of the poorest IC, and a minimum of 0, denoting optimal IC. The categorical variable was transformed to create discrete score, categorized as 0, 1, 2, 3, or 4+ score.

**eTable 16 Sensitivity analysis of the associations between IC and risk of depression, anxiety, and depression/anxiety after excluding participants with baseline depressive symptoms**

| **IC deficit scores** | **N_case_/N_participants_** | **HR (95% CI)** | ***P* for trend** |
| --- | --- | --- | --- |
| **Depression** | |  | <0.001 |
| 0 (ref, better IC) | 3359/130878 | Reference |  |
| 1 | 4786/135929 | 1.35 (1.29-1.41) | |
| 2 | 3250/68725 | 1.73 (1.65-1.82) | |
| 3 | 1644/23214 | 2.44 (2.29-2.59) | |
| 4+ | 859/7813 | 3.48 (3.21-3.76) | |
| **Anxiety** |  |  | <0.001 |
| 0 (ref, better IC) | 3848/130878 | Reference |  |
| 1 | 5217/135929 | 1.30 (1.24-1.35) | |
| 2 | 3255/68725 | 1.56 (1.49-1.63) | |
| 3 | 1501/23214 | 2.05 (1.92-2.18) | |
| 4+ | 699/7813 | 2.71 (2.49-2.95) | |
| **Depression/Anxiety** | |  | <0.001 |
| 0 (ref, better IC) | 6222/130878 | Reference |  |
| 1 | 8508/135929 | 1.30 (1.26-1.35) | |
| 2 | 5525/68725 | 1.62 (1.57-1.69) | |
| 3 | 2633/23214 | 2.19 (2.09-2.30) | |
| 4+ | 1254/7813 | 2.94 (2.76-3.14) | |

HR, hazard ratio; CI, confidence interval; IC, intrinsic capacity.

The model was adjusted for age, sex, ethnicity, Townsend deprivation index, education level, annual before-tax household income, BMI, smoking status, alcohol intake frequency, physical activity, disease histories of hypertension status, diabetes status and cardiovascular disease status.

IC scores were assessed by seven factors that reflected functional status to compute a comprehensive IC deficit score, yielding a potential maximum of 7, indicative of the poorest IC, and a minimum of 0, denoting optimal IC. The categorical variable was transformed to create discrete scores, categorized as 0, 1, 2, 3, or 4+ scores.

**eTable 17 Sensitivity analyses for the association between IC and risk of depression, anxiety and depression/anxiety when excluding events occurring within the first 2 years of follow-up**

| **IC deficit score** | **N_case_/N_total_** | **HR (95% CI)** | ***P* for trend** |  |
| --- | --- | --- | --- | --- |
|  |  |  |  |  |
| **Depression** |  |  | <0.001 |  |
| 0 (ref, better IC) | 3644/140661 | Reference |  |  |
| 1 | 5487/150411 | 1.35 (1.28-1.42) |  |  |
| 2 | 4054/79951 | 1.78 (1.68-1.88) |  |  |
| 3 | 2257/29404 | 2.41 (2.25-2.59) |  |  |
| 4+ | 1319/11094 | 3.45 (3.15-3.76) |  |  |
| **Anxiety** |  |  | <0.001 |  |
| 0 (ref, better IC) | 4670/144422 | Reference |  |  |
| 1 | 6771/156591 | 1.32 (1.26-1.38) |  |  |
| 2 | 4852/85115 | 1.66 (1.58-1.75) |  |  |
| 3 | 2575/32409 | 2.25 (2.11-2.40) |  |  |
| 4+ | 1507/13243 | 2.96 (2.73-3.21) |  |  |
| **Depression/Anxiety** |  |  | <0.001 |  |
| 0 (ref, better IC) | 6131/137405 | Reference |  |  |
| 1 | 8758/146337 | 1.31 (1.26-1.36) |  |  |
| 2 | 6121/77332 | 1.66 (1.59-1.74) |  |  |
| 3 | 3170/28171 | 2.24 (2.12-2.37) |  |  |
| 4+ | 1700/10533 | 2.95 (2.74-3.18) |  |  |

HR, hazard ratio; CI, confidence interval; IC, intrinsic capacity.

The model was adjusted for age, sex, ethnicity, Townsend deprivation index, education level, annual before-tax household income, BMI, smoking status, alcohol intake frequency, physical activity, disease histories of hypertension status, diabetes status and cardiovascular disease status.

IC scores were assessed by seven factors that reflected functional status to compute a comprehensive IC deficit score, yielding a potential maximum of 7, indicative of the poorest IC, and a minimum of 0, denoting optimal IC. The categorical variable was transformed to create discrete scores, categorized as 0, 1, 2, 3, or 4+ scores.

**eTable 18 Sensitivity analyses for the association between IC and risk of depression, anxiety and depression/anxiety when excluding events occurring within the first 5 years of follow-up**

| **IC deficit scores** | **N_case_/N_participants_** | **HR (95% CI)** | ***P* for trend** |
| --- | --- | --- | --- |
| **Depression** |  |  | <0.001 |
| 0 (ref, better IC) | 2550/136079 | Reference |  |
| 1 | 3808/144475 | 1.36 (1.29-1.43) | |
| 2 | 2708/75938 | 1.74 (1.65-1.84) | |
| 3 | 1477/27400 | 2.42 (2.26-2.59) | |
| 4+ | 849/10104 | 3.40 (3.13-3.70) | |
| **Anxiety** |  |  | <0.001 |
| 0 (ref, better IC) | 3047/136079 | Reference |  |
| 1 | 4328/144475 | 1.32 (1.26-1.38) | |
| 2 | 2884/75938 | 1.62 (1.53-1.70) | |
| 3 | 1413/27400 | 2.10 (1.96-2.24) | |
| 4+ | 718/10104 | 2.75 (2.52-2.99) | |
| **Depression/Anxiety** | 12390/393996 | | <0.001 |
| 0 (ref, better IC) | 4805/136079 | Reference |  |
| 1 | 6896/144475 | 1.32 (1.27-1.37) | |
| 2 | 4727/75938 | 1.65 (1.58-1.72) | |
| 3 | 2399/27400 | 2.19 (2.08-2.30) | |
| 4+ | 1271/10104 | 2.94 (2.75-3.13) | |

HR, hazard ratio; CI, confidence interval; IC, intrinsic capacity.

The model was adjusted for age, sex, ethnicity, Townsend deprivation index, education level, annual before-tax household income, BMI, smoking status, alcohol intake frequency, physical activity, disease histories of hypertension status, diabetes status and cardiovascular disease status.

IC scores were assessed by seven factors that reflected functional status to compute a comprehensive IC deficit score, yielding a potential maximum of 7, indicative of the poorest IC, and a minimum of 0, denoting optimal IC. The categorical variable was transformed to create discrete scores, categorized as 0, 1, 2, 3, or 4+ scores.

**eTable 19 Sensitivity analyses for the association between IC deficit score and risk of depression, anxiety and depression/anxiety after excluding the psychological domain from the IC score**

| **IC deficit scores** | **N_case_/N_participants_** | **HR (95% CI)** | ***P* for trend** |
| --- | --- | --- | --- |
| **Depression** | |  | <0.001 |
| 0 (ref, better IC) | 7053/202797 | Reference |  |
| 1 | 7395/154862 | 1.34 (1.30-1.39) | |
| 2 | 3678/55382 | 1.74 (1.67-1.82) | |
| 3 | 1314/12678 | 2.45 (2.30-2.61) | |
| 4+ | 243/1892 | 2.83 (2.48-3.23) | |
| **Anxiety** |  |  | <0.001 |
| 0 (ref, better IC) | 8657/209996 | Reference |  |
| 1 | 8741/162589 | 1.28 (1.25-1.32) | |
| 2 | 4164/59414 | 1.58 (1.52-1.64) | |
| 3 | 1423/14125 | 2.13 (2.01-2.26) | |
| 4+ | 263/2252 | 2.39 (2.11-2.71) | |
| **Depression/Anxiety** | |  | <0.001 |
| 0 (ref, better IC) | 11478/198128 | Reference |  |
| 1 | 11390/150754 | 1.28 (1.25-1.32) | |
| 2 | 5367/53661 | 1.61 (1.55-1.66) | |
| 3 | 1796/12225 | 2.20 (2.08-2.31) | |
| 4+ | 312/1798 | 2.47 (2.20-2.77) | |

HR, hazard ratio; CI, confidence interval; IC, intrinsic capacity.

The model was adjusted for age, sex, ethnicity, Townsend deprivation index, education level, annual before-tax household income, BMI, smoking status, alcohol intake frequency, physical activity, disease histories of hypertension status, diabetes status and cardiovascular disease status.

The IC deficit score was recalculated after excluding the psychological domain, which comprised self-reported exhaustion and sleep duration, and was categorized as 0, 1, 2, 3, and 4+.

**eTable 20 Sensitivity analyses for the association between IC and risk of depression, anxiety and depression/anxiety when excluding all participants without completed covariates**

| **IC deficit scores** | **N_case_/N_participants_** | **HR (95% CI)** | ***P* for trend** |
| --- | --- | --- | --- |
| **Depression** |  |  | <0.001 |
| 0 (ref, better IC) | 2921/104889 | Reference |  |
| 1 | 4181/107854 | 1.36 (1.30-1.43) |  |
| 2 | 2978/54298 | 1.84 (1.75-1.94) |  |
| 3 | 1497/18493 | 2.50 (2.35-2.67) |  |
| 4+ | 845/6367 | 3.63 (3.35-3.95) |  |
| **Anxiety** |  |  | <0.001 |
| 0 (ref, better IC) | 3597/107815 | Reference |  |
| 1 | 5018/112305 | 1.33 (1.27-1.38) |  |
| 2 | 3350/57700 | 1.67 (1.59-1.75) |  |
| 3 | 1723/20412 | 2.29 (2.16-2.44) |  |
| 4+ | 926/7636 | 3.01 (2.78-3.25) |  |
| **Depression/Anxiety** |  |  | <0.001 |
| 0 (ref, better IC) | 4861/102733 | Reference |  |
| 1 | 6656/105274 | 1.32 (1.27-1.37) |  |
| 2 | 4406/52696 | 1.69 (1.62-1.77) |  |
| 3 | 2148/17847 | 2.31 (2.19-2.43) |  |
| 4+ | 1080/6098 | 3.11 (2.90-3.34) |  |

HR, hazard ratio; CI, confidence interval; IC, intrinsic capacity.

The model was adjusted for age, sex, ethnicity, Townsend deprivation index, education level, annual before-tax household income, BMI, smoking status, alcohol intake frequency, physical activity, disease histories of hypertension status, diabetes status and cardiovascular disease status.

IC scores were assessed by seven factors that reflected functional status to compute a comprehensive IC deficit score, yielding a potential maximum of 7, indicative of the poorest IC, and a minimum of 0, denoting optimal IC. The categorical variable was transformed to create discrete scores, categorized as 0, 1, 2, 3, or 4+ scores.

**eTable 21 Sensitivity analyses for the association between IC and risk of depression, anxiety, and depression/anxiety when excluding BMI**

| **IC deficit scores** | **Ncase/Nparticipants** | **HR (95% CI)** | ***P* for trend** |
| --- | --- | --- | --- |
| **Depression** |  |  | <0.001 |
| 0 (ref, better IC) | 3715/138098 | Reference |  |
| 1 | 5606/147369 | 1.41 (1.36-1.47) | |
| 2 | 4189/78119 | 1.96 (1.87-2.05) | |
| 3 | 2326/28619 | 2.86 (2.71-3.02) | |
| 4+ | 1389/10813 | 4.35 (4.08-4.64) | |
| **Anxiety** |  |  | <0.001 |
| 0 (ref, better IC) | 4216/138098 | Reference |  |
| 1 | 5933/147369 | 1.32 (1.26-1.37) | |
| 2 | 4026/78119 | 1.65 (1.58-1.73) | |
| 3 | 2049/28619 | 2.23 (2.11-2.35) | |
| 4+ | 1078/10813 | 3.03 (2.83-3.25) | |
| **Depression/Anxiety** |  |  | <0.001 |
| 0 (ref, better IC) | 6824/138098 | Reference |  |
| 1 | 9790/147369 | 1.35 (1.31-1.39) | |
| 2 | 6908/78119 | 1.77 (1.71-1.83) | |
| 3 | 3618/28619 | 2.47 (2.37-2.58) | |
| 4+ | 1980/10813 | 3.52 (3.34-3.71) | |

HR, hazard ratio; CI, confidence interval; IC, intrinsic capacity.

The model was adjusted for age, sex, ethnicity, Townsend deprivation index, education level, annual before-tax household income, smoking status, alcohol intake frequency, physical activity, disease histories of hypertension status, diabetes status and cardiovascular disease status.

IC scores were assessed by seven factors that reflected functional status to compute a comprehensive IC deficit score, yielding a potential maximum of 7, indicative of the poorest IC, and a minimum of 0, denoting optimal IC. The categorical variable was transformed to create discrete scores, categorized as 0, 1, 2, 3, or 4+ scores.

**eTable 22 Sensitivity analysis of the associations between IC and risk of depression, anxiety, and depression/anxiety after additional adjustment for television viewing time and computer use time**

| **IC deficit scores** | **N_case_/N_participants_** | **HR (95% CI)** | ***P* for trend** |
| --- | --- | --- | --- |
| **Depression** | |  | <0.001 |
| 0 (ref, better IC) | 3715/138098 | Reference |  |
| 1 | 5606/147369 | 1.39 (1.33-1.45) | |
| 2 | 4189/78119 | 1.86 (1.78-1.95) | |
| 3 | 2326/28619 | 2.60 (2.46-2.75) | |
| 4+ | 1389/10813 | 3.71 (3.47-3.97) | |
| **Anxiety** |  |  | <0.001 |
| 0 (ref, better IC) | 4216/138098 | Reference |  |
| 1 | 5933/147369 | 1.30 (1.25-1.36) | |
| 2 | 4026/78119 | 1.62 (1.55-1.70) | |
| 3 | 2049/28619 | 2.14 (2.03-2.26) | |
| 4+ | 1078/10813 | 2.85 (2.65-3.06) | |
| **Depression/Anxiety** | |  | <0.001 |
| 0 (ref, better IC) | 6824/138098 | Reference |  |
| 1 | 9790/147369 | 1.33 (1.29-1.37) | |
| 2 | 6908/78119 | 1.71 (1.65-1.77) | |
| 3 | 3618/28619 | 2.31 (2.21-2.41) | |
| 4+ | 1980/10813 | 3.13 (2.97-3.31) | |

IC, intrinsic capacity; HR, hazard ratio; CI, confidence interval.

The model was additionally adjusted for television viewing time and computer use time, on top of the covariates included in the fully adjusted model.

**eTable 23 Sensitivity analysis of the associations between IC and risk of depression, anxiety, and depression/anxiety after additional adjustment for baseline psychotropic medication use and chronic pain**

| **IC deficit scores** | **N_case_/N_participants_** | **HR (95% CI)** | ***P* for trend** |
| --- | --- | --- | --- |
| **Depression** |  |  | <0.001 |
| 0 (ref, better IC) | 3715/138098 | Reference |  |
| 1 | 5606/147369 | 1.35 (1.29-1.41) |  |
| 2 | 4189/78119 | 1.74 (1.67-1.82) |  |
| 3 | 2326/28619 | 2.21 (2.09-2.33) |  |
| 4+ | 1389/10813 | 2.72 (2.54-2.91) |  |
| **Anxiety** |  |  | <0.001 |
| 0 (ref, better IC) | 4216/138098 | Reference |  |
| 1 | 5933/147369 | 1.54 (1.48-1.61) |  |
| 2 | 4026/78119 | 1.90 (1.80-2.01) |  |
| 3 | 2049/28619 | 2.23 (2.07-2.40) |  |
| 4+ | 1078/10813 | 2.20 (1.96-2.47) |  |
| **Depression/Anxiety** |  |  | <0.001 |
| 0 (ref, better IC) | 6824/138098 | Reference |  |
| 1 | 9790/147369 | 1.30 (1.26-1.34) |  |
| 2 | 6908/78119 | 1.62 (1.56-1.67) |  |
| 3 | 3618/28619 | 2.01 (1.93-2.10) |  |
| 4+ | 1980/10813 | 2.39 (2.26-2.53) |  |

HR, hazard ratio; CI, confidence interval; IC, intrinsic capacity.

The model was additionally adjusted for baseline psychotropic medication use and chronic pain, on top of the covariates included in the fully adjusted model.

IC scores were assessed by seven factors that reflected functional status to compute a comprehensive IC deficit score, yielding a potential maximum of 7, indicative of the poorest IC, and a minimum of 0, denoting optimal IC. The categorical variable was transformed to create discrete scores, categorized as 0, 1, 2, 3, or 4+ scores.

**eTable 24 Incremental predictive value of IC beyond the conventional model for risk of depression, anxiety, and depression/anxiety**

| **Outcome** | **Conventional model (Reference) C-index (95% CI)** | **Conventional model + IC C-index (95% CI)** | **ΔC-index** | **NRI (95% CI)** | ***P* value** | **IDI (95% CI)** | ***P* value** |
| --- | --- | --- | --- | --- | --- | --- | --- |
| Depression | 0.643 (0.639–0.647) | 0.669 (0.665–0.673) | 0.026 | 0.107 (0.080–0.139) | <0.001 | 0.010 (0.006–0.014) | <0.001 |
| Anxiety | 0.619 (0.615–0.623) | 0.638 (0.634–0.643) | 0.019 | 0.050 (0.016–0.090) | <0.001 | 0.004 (0.001–0.006) | <0.001 |
| Depression/Anxiety | 0.626 (0.622–0.629) | 0.648 (0.645–0.651) | 0.022 | 0.076 (0.041–0.103) | <0.001 | 0.008 (0.004–0.010) | <0.001 |

IC, intrinsic capacity; CI, confidence interval; NRI, net reclassification improvement; IDI, integrated discrimination improvement.

Conventional model included age, sex, ethnicity, Townsend deprivation index, BMI, education level, annual before-tax household income, smoking status, alcohol intake frequency, physical activity, disease histories of hypertension status, diabetes status and cardiovascular disease status.

**eFigure 1 The flowchart of participants selection**

**eFigure 2 Cumulative hazard curve of depression, anxiety and depression/anxiety according to IC deficit scores**

X-axis: Time (years), Y-axis: Cumulative probability of developing depression, anxiety and depression/anxiety. Each curve represents a group based on intrinsic capacity, specifically groups 0, 1, 2, 3, and 4+.

The number of risk events occurring in each time period is shown below.

**eFigure 3 Association between IC deficit score and the score of each item of PHQ-9 and GAD-7**

Abbreviations: BMI, body mass index; OR, odds ratio; IC, intrinsic capacity; PHQ-9, Patient Health Questionnaire-9; GAD-7, Generalized Anxiety Disorder-7.

Model 1 was not adjusted.

Model 2 was adjusted for age, sex, and ethnicity.

Model 3 was adjusted for age, sex, ethnicity, Townsend deprivation index, education level, annual before-tax household income, BMI, smoking status, alcohol intake frequency, physical activity, disease histories of hypertension status, diabetes status and cardiovascular disease status.

**References**

1.	Ramírez-Vélez R, Borda MG, Sáez de Asteasu ML, Izquierdo M. Intrinsic capacity as a predictor of depression onset in middle-aged and older adults: Insights from the UK Biobank. *J Affect Disord* (2025) 388:119590. doi: 10.1016/j.jad.2025.119590

2.	Cullen B, Nicholl BI, Mackay DF, Martin D, Ul-Haq Z, McIntosh A, Gallacher J, Deary IJ, Pell JP, Evans JJ, et al. Cognitive function and lifetime features of depression and bipolar disorder in a large population sample: Cross-sectional study of 143,828 UK Biobank participants. *Eur Psychiat* (2015) 30:950–958. doi: 10.1016/j.eurpsy.2015.08.006

3.	Tian J, Jones G, Lin X, Zhou Y, King A, Vickers J, Pan F. Association between chronic pain and risk of incident dementia: findings from a prospective cohort. *BMC Med* (2023) 21:169. doi: 10.1186/s12916-023-02875-x
